# Implementation of a soft grading system for chemistry in a Moodle plugin: reaction handling

**DOI:** 10.1186/s13321-024-00889-y

**Published:** 2024-08-01

**Authors:** Louis Plyer, Gilles Marcou, Céline Perves, Fanny Bonachera, Alexander Varnek

**Affiliations:** 1 Faculté de Chimie, University of Strasbourg, Strasbourg, France; 2 Laboratory of Chemoinformatics-UMR7140, University of Strasbourg, Strasbourg, France; 3 Direction du Numérique (DNUM), University of Strasbourg, Strasbourg, France

**Keywords:** Educational chemistry, Softgrading, Moodle, Plugin, Chemical reactions

## Abstract

Here, we present a new method for evaluating questions on chemical reactions in the context of remote education. This method can be used when binary grading is not sufficient as some tolerance may be acceptable. In order to determine a grade, the developed workflow uses the pairwise similarity assessment of two considered reactions, each encoded by a single molecular graph with the help of the Condensed Graph of Reaction (CGR) approach. This workflow is part of the ChemMoodle project and is implemented as a Moodle Plugin. It uses the Chemdoodle engine for reaction drawing and visualization and communicates with a REST server calculating the similarity score using ISIDA fragment descriptors. The plugin is open-source, accessible in GitHub (https://github.com/Laboratoire-de-Chemoinformatique/moodle-qtype_reacsimilarity) and on the Moodle plugin store (https://moodle.org/plugins/qtype_reacsimilarity?lang=en). Both similarity measures and fragmentation can be configured.

**Scientific contribution**

This work introduces an open-source method for evaluating chemical reaction questions within Moodle using the CGR approach. Our contribution provides a nuanced grading mechanism that accommodates acceptable tolerances in reaction assessments, enhancing the accuracy and flexibility of the grading process.

## Introduction

Numerous studies have demonstrated that the completion of homework activities exerts a substantial influence on the academic achievement of individual students [1, 2]. Besides exerting an impact on the grades of the students, homework activities, particularly those conducted online, are viewed by students as valuable learning tools [3]. Thus, Vijay S. Vyas and Scott A. Reid concluded that “a combination of active learning pedagogy, core concepts curricula, and incorporation of low-stakes assessments is a strategy capable of moving the needle to improve DFW rates in second-term general chemistry” [4] where DFW rates are the % of D and F grades and withdrawals in a given class. This emphasizes the utility for low-stakes assessments and the need of technical tools to implement them. The importance of distance learning in Chemistry has increased considerably in response to the Covid 19-health crisis [5]. Yet, distance learning has been a long-time concern for the modernization of pedagogy. It is exemplified in the “Charte de l’Enseignement à Distance” [6] of the University of Strasbourg. This implies the development of questionnaires for which correction is automated. These questionnaires are used during the registration of new students in order to identify knowledge gaps and skill deficiencies [7]. They also serve as an instrument of formative assessment in the preparation and consolidation of knowledge, enabling learners to identify their strengths and weaknesses, benefiting from instructors’ feedback, and adjusting their learning strategies [8].

Hence, the existence of tools facilitating online homework assignments in the domain of chemistry is natural and several authors have proposed operational solutions. Thus, Korsakova et al. [9] described a “Chemist Bot” helping Russian students to prepare for chemistry exams. It is designed as a conversational bot proposing remediation articles and text-based quiz questions with immediate feedback. In general, students who used the Chemist Bot performed better on the United State Exam (USE) of the Russian Federation.

O’Sullivan and Hargarden reported one of the earliest instances of utilizing a chemical structure sketcher in online tutorials with automated correction [10]. The students’ drawings are converted into a canonical SMILES (Simplified Molecular Input Line Entry Specification) [11] string, which are then assessed by comparing them to an anticipated response also in SMILES format. This approach is implemented in the SOCOT platform, which is overseen by the University of Cork and the Dublin Institute of Technology. Similarly, Otálvaro has proposed a method [12] that involves a sequential procedure for students to respond to chemical questions utilizing the JSME editor [13] on their mobile devices. The students are required to generate the SMILES notation of their answer, which they have to copy and paste into ChemDrawJS [14] to generate an InChI [15] code. Ultimately, the InChI code must be pasted into Socrative [16], the learning management system (LMS) / web-based classroom response system (WBCRS) used in that contribution.

Earlier, we proposed the implementation of a soft grading system for chemistry in the Moodle platform [17] able to automatically evaluate the candidates whose answers contain a chemical structure drawing. This method proposes a mark for the answer proportional to the graph similarity between the answer and the solution. Moodle is the most widely used free LMS [18].

In contrast to “multiple-choice” (closed-ended) questions, this method allows for “constructed-response” (open-ended) questions. This change of methodology makes the self-assessment more effective, as it insists on reasoning instead of trails and errors efforts. [19]. Indeed, as estimated by Richard-Babb et al. [20] about closed-ended questions, approximately one-third of students resort to attempting different suggested options instead of referring to course materials and reasoning when faced with difficult questions.

Liu et al. [21] discussed the advantages and disadvantages of both open-ended and closed-ended question types. Closed-ended questions have low variability in their grading as opposed to the variability originating from the teachers correcting open-ended questions. Open-ended questions typically require more time to score. Yet open-ended question enables for a direct assessment of the students’ knowledge without any help/clues: it enables the teachers to identify possible students' misconceptions and inconsistencies. The analysis of the answers to open-ended questions is valuable for the creation of more effective teaching and remediation actions.

The ChemMoodle plugins combining *Reacsimilarity* (described here), *Molsimilarity* [17], and *MolStructure* [22] tools allow the teachers to benefit from strong points of both open-ended and closed-ended strategies mentioned by Liu et al. [21]: easy, fast, objective, and reproducible scoring while retaining the ability to highlight and understand the possible misconceptions from the students.

A few tools allowing teachers to ask questions about chemical reactions were reported in the literature [10, 23]. As an answer, the students are supposed either to draw a single molecule [10] or to write a reaction equation [23]. In the last accessible versions of the plugin [24] (before its replacement by the OpenOChem platform [23]), the correction of the reaction equation was done by comparison of expected and students’ SMILES of the reaction. The exact fit between expected and students answers was used for the automated assessment process in either case.

In this work, we propose a new approach based on the Condensed Graph of Reaction (CGR) [25–28] method. This technique allows to compare the students’ answers concerning chemical reaction equation to the expected answer from the teachers in a way analogous to the Molsimilarity plugin. To our knowledge, it is the only completely open-source chemical question type plugin to work with reactions, while guaranteeing privacy—the plugin does not trigger any undesired communication over the internet. In the following, we discuss implementation details and how user feedback has been taken into account. Feedbacks were gathered during 5 hackathons organized by the University of Strasbourg between September 2023 and May 2024 to present the tools to end users, mostly teachers. The plugin has been released on the Moodle store in November 2023. As of May 2024, four sites are using it.

### CGR/atom mapping

A CGR [25–28] allows to encode a chemical reaction by a single molecular graph. A CGR is described by both conventional chemical bonds (single, double, triple, aromatic) and so-called dynamic bonds and dynamic atoms characterizing chemical transformations. Thus, dynamic bonds describe breaking, formation, and changing bond order whereas dynamic atoms describe the change of their oxidation number as a result of the chemical reaction. CGR results from superimposing reactants’ and products’ atoms bearing the same identifiers. Earlier [25–28], the CGR approach was used in the building of machine-learning models for the rate constant of SN2 reactions [28], reaction similarity searches [29], protective groups’ reactivity assessment [30], and reaction condition prediction [31]. In conjunction with an autoencoder, it has also been used for an AI-based generation of chemical reactions [32].

**Fig. 1:**
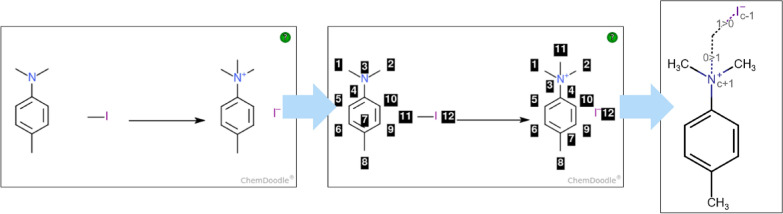
Atom mapping process: Unmapped reaction on the left, atom mapping of the reaction in the middle and associated CGR on the right (“0 > 1”: create a single bond; “1 > 0”: break a single bond)

The superposition of atoms of reactants and products to construct the CGR requires atom-to-atom mapping (AAM) (Fig. 1 shows the process for the reaction with CAS Number 31-031-CAS-23647760). It consists in assigning to each reactant’s atom a unique number and the same unique number to the corresponding atom in the products. AAM is a valuable tool for the classification of chemical reactions and elucidation of the reactions’ mechanisms. AAM can be associated to a pattern matching exercise and is usually performed algorithmically [33]. However, these patterns are ambiguous. A proper formulation of the electronic reorganization over the reactants disambiguates the matched patterns, leading to a specific AAM. To enable students to gain and test their understanding of chemical reactions, they are asked to manually number each atom in the reactants and products.

To shed light on reaction mechanisms by describing electrons movement, the teachers can use curved arrow notations (“mechanistic” arrows). Houchlei et al. [34] have raised the point that students understanding a mechanism, with the help of mechanistic arrows, are more efficient when faced to a new mechanism compared to students learning by heart.

Quiz questions involving mechanistic arrows are covered by the OpenOChem [23] tools, which is a different and complementary approach compared to the *Reacsimilarity* module. In *ReacSimilarity*, the correct mapping results from a correct understanding of the electron movements. We believe that by performing AAM manually, the students can effectively track the movement of electrons, capture reorganization processes, and challenge their understanding of chemical reactivity [35]. In such a way, this is a valuable didactic exercise, providing students with a practical experience of elucidation of reaction mechanisms at the atomic level. To define one AAM, the user selects the “Reaction mapping” arrow in the arrow toolset, then click on a reactant atom and drags to the corresponding product atom. The exercise can be tedious for a student if they have to start from scratch. However, by using “reaction templates”, the teacher can guide the students to specific parts of a reaction to be evaluated. For instance, the teacher may map some of the atom pairs in advance. This reduces the amount of input needed from the students, allowing them to answer faster. For a given reaction, alternative correct AAM are possible. All of them correspond to the same CGR.

### ISIDA fragment descriptors

Once the CGRs are built from the reactions drawn by both students and teachers, they are encoded by the ISIDA fragment descriptors [36] generated for the 2D molecular graph to compute the grades, similarly to the *Molsimilarity* module. There are 3 types of ISIDA descriptors: (i) sequences of atoms and bonds or atoms only, (ii) atom-centered fragments, and (iii) pharmacophoric triplets. In such a way, a molecular graph can be encoded by a vector consisting of the fragments’ counts.

### Implementation

**Fig. 2:**
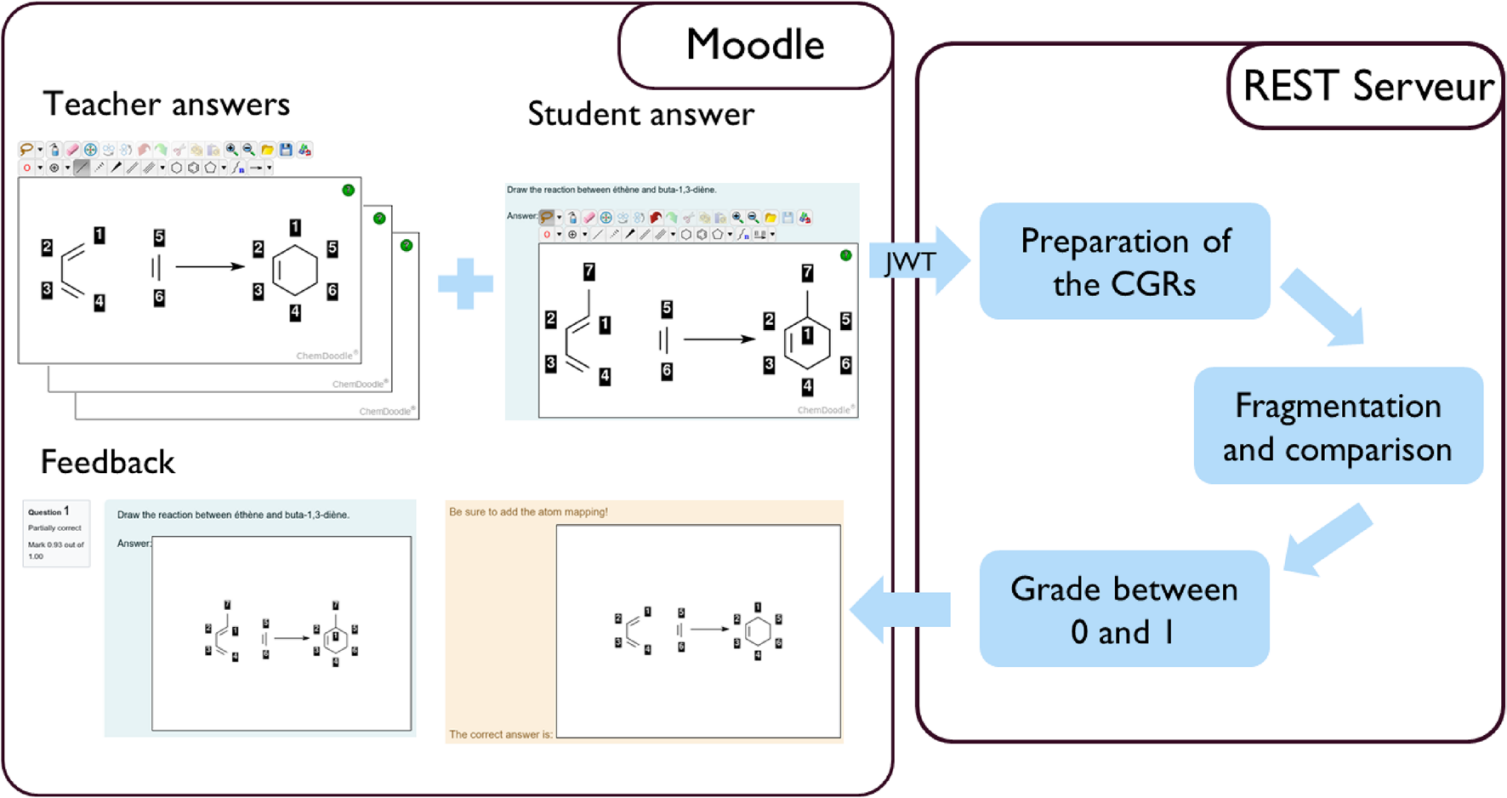
Workflow of the plugin. The teacher prepares a question and the student an answer (left panel “Moodle”). Both are sent to a server (right panel “REST Server”) where they are interpreted and compared on a 0 (completely different) to 1 (complete match) scale. This estimate of the grade is sent back to the student and teacher for feedback and assessment purposes (left panel “Moodle”)

The workflow of the *Reacsimilarity* plugin is given in Fig. 2. Although it is similar to the *Molsimilarity* module [17], some differences should be pointed out. The user interface on the Moodle side uses Chemdoodle Web Component [37], and exports both the RXN (reaction data file) and Chemdoodle JSON format inside a JSON (JavaScript Object Notation) [38] to the Moodle database. The Chemdoodle code was amended to append the atom-to-atom mapping to the RXN, and to be able to show AAM in the feedback process. The communication between Moodle and the REST server is secured by the JSON Web Token (JWT) [39] standard. The exchange of data between Moodle and the Rest server is based on the JSON format.

**Fig. 3:**
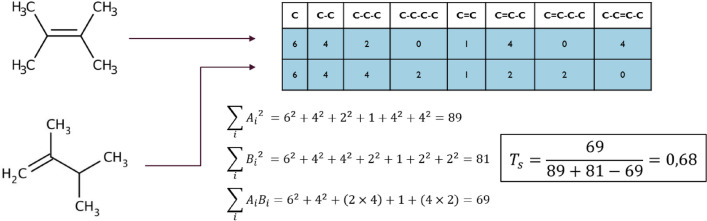
Tanimoto similarity calculation, with ISIDA descriptors. The formula used is Formula 1

A REST server is used for the assessment of similarity between the students’ and teachers’ structures. First, the reactant and product molecules are aromatized with the help of the Indigo library [40]. The REST server transforms the reaction to CGR using ISIDA software package [41]. ISIDA fragments descriptors and pairwise Tanimoto similarity are then computed by the correction server. The ISIDA descriptors are computed using the ISIDA Fragmentor2022 tool [36] (Fig. 3). The whole process is implemented as a REST API and hosted on a REST server. The latter can be launched from the same server as Moodle and can be encapsulated in a virtual machine or in a docker container managed by a RabbitMQ system [42]. This server communicates exclusively with the Moodle server.

The fragmentation scheme of the CGRs is defined by default but can be modified by the administrator of the correction server by amending a set of parameters stored in the configuration file in XML format. By modifying these parameters, the user can change the size of the descriptors, the sensitivity to bond types, atom types, or both. The documentation of the plugin is describing which configuration file to edit and how. The molecular descriptors used for the correction of a given question are computed on the fly using the chemical structures input of both the students and the teachers. The ISIDA molecular descriptors (ISIDA fragments) support the encoding of radicals, lone pairs, and formal charges. Presence or absence of explicit hydrogens have an impact on the encoding of the answers and the solution to a quiz question. Therefore, teachers must provide instructions on how they expect the drawings to be done and provide alternative answers with and without hydrogens, when deemed needed. These alternative answers can be added a posteriori if necessary. Although the chemistry sketcher represents implicit hydrogens, those cannot be mapped, preventing any possible confusion.

**Fig. 4:**
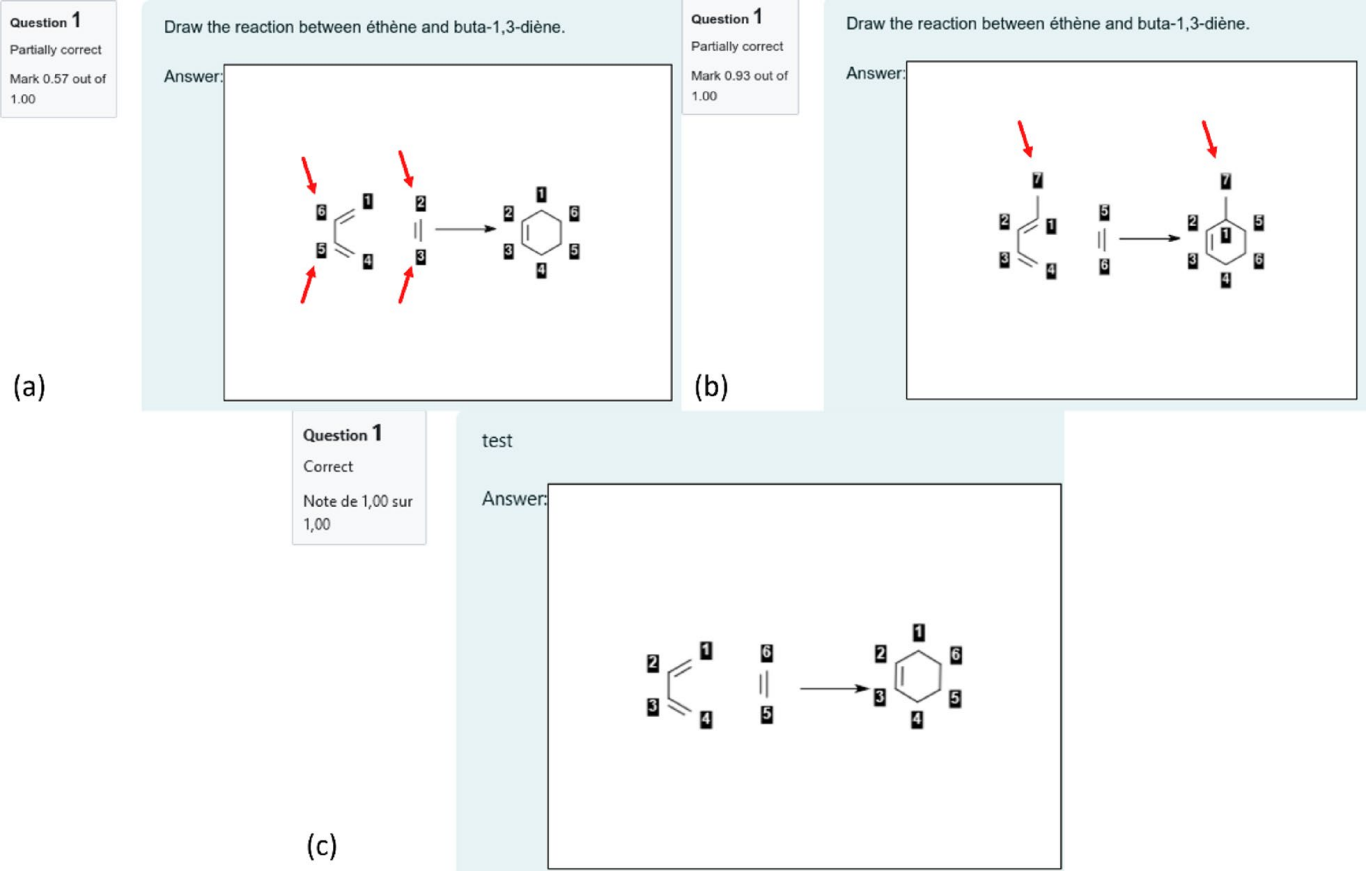
*Top*: Two erroneous students answers concerning the Diels Alder reaction between ethylene and buta-1,3-diène related to (**a**) wrong mapping of atoms in reaction center which provides a score of 0.57 and (**b**) addition of the Me group to butadiene providing a score of 0.93. The red arrows emphasize on the errors made by the students. **c** Expected answer

As mentioned before, the students perform the AAM needed for CGR construction. The teachers can provide students with a starting point using the "reaction template", a partial drawing of the reaction that may include a partial atom-to-atom mapping. This partial AAM is useful to guide the evaluation to focus on a specific part of the reaction only. This mapping procedure is not time-consuming. Any mistakes in AAM results in the creation of wrong dynamic bonds. Notice that an error on the reaction center has more impact than an error on other parts of the molecules. Indeed, an error on the reaction center would modify the type of dynamic atoms and bonds, and therefore modify molecular fragments. As consequence, the CGR fragments descriptor vectors of the correct and erroneous answers are orthogonal. On the other hand, a modification out of the reaction center may still lead to CGR fragments that are in common between the correction and the answer. This is exemplified in Fig. 4 where an error on the reaction center of the Diels Alder reaction between ethylene and buta-1,3-diene (wrong AAM, Fig. 4a) yields a score of 0.57, while an incorrect nucleophile (penta-1,3-diene instead of buta-1,3-diene, Fig. 4b) yields a score of 0.93.

Stereochemistry analysis is performed with the help of the InChI [15] strings generated with the InChI v.1.06 program. We chose to use InChI as the stereochemistry information is located in defined layers that are technically simple to compare. The InChI strings are computed for reactants and products of both students’ and teachers’ answers. Then, the correction server compares the information in the InChI stereo-layers [/t “stereo labels” on atoms, /m and /s complementary “chirality label”], where “stereo label” = “+” and “−” and “chirality label” = “0” and “1”.

## Results

**Fig. 5:**
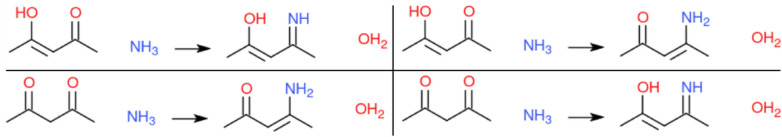
Alternative correct answers for the Pentane-2,4-dione with ammonia reaction, taking in account the tautomeric structures

For a given question, a teacher prepares from 1 to N reaction equations that are considered correct. It allows to accommodate for chemical ambiguities (mesomeres, tautomers, etc.), see Fig. 5 for an example of the reaction between Pentane-2,4-dione and Ammonia. It also adds some degrees of freedom to the teachers concerning the questions that may be asked.

A teacher can incorporate an initial “reaction template” representing a part of reaction equation (e.g., reactants or products only) or full reaction equation with or without AAM. The student is supposed to finalize reaction equation and to add the mapping. This allows teacher to focus the test on more specific skills and knowledge, such as reaction mechanisms questions. This feature has also been integrated into the most recent version of the Molsimilarity plugin.

For each of these answers, the teachers will draw the reaction using the Chemdoodle Web sketcher, map the reaction (Fig. 1, middle) and click on the “Insert given reaction as answer / update the answer with the reaction”. To map the reaction, the user must use the “Reaction Mapping” tool, then click and drag from one atom of the reactant to one atom of the products. Both Chemdoodle JSON format and RXN files will be stored in JSON format [38] in the Moodle database. In addition to chemical reactions, the teachers have the option to provide instructions and feedback for the students.

There are two types of feedback available: “general” feedback that is not related to grades, and “specific” feedback that is displayed when a grade is below 1. The specific feedback aims to assist students in improving their responses.

When taking a test, students follow the instructions and draw the required chemical reaction. Upon test completion, the students’ answers are processed, following the same procedure as the teachers’ answers. Then, the students’ and teachers’ answers are sent to the REST API through a cURL [43] request and authenticated via JWT standard. The similarity is then computed by the REST API. The REST API is written in Pascal Object language [44], enabling support of a large number of computer systems at binary code and trivial recompilation from the source—for those interested.

If the server fails to respond to the request, a warning message is sent to the Moodle administrator. In this case, the students’ answers will be saved and flagged as “needing correction”. If an unauthenticated request is made, the Moodle administrator will be warned too, and the IP address of the attacker will be linked in the corresponding warning.

As mentioned above, to compute the grade, reactions are aromatized using the Indigo library [40]. The CGR of the reactants and products from the students and teachers are generated using AAM. Then, both the students’ and teachers’ CGRs are encoded using the ISIDA molecular descriptors. Fragmentation IAB (2–4) FC_UR is used, standing for sequences of 2–4 atoms and bonds, considering formal charges, lone pairs, and radicals. The configuration of the fragmentation is stored in an XML file that can be edited by the Moodle administrator.

**Fig. 6:**
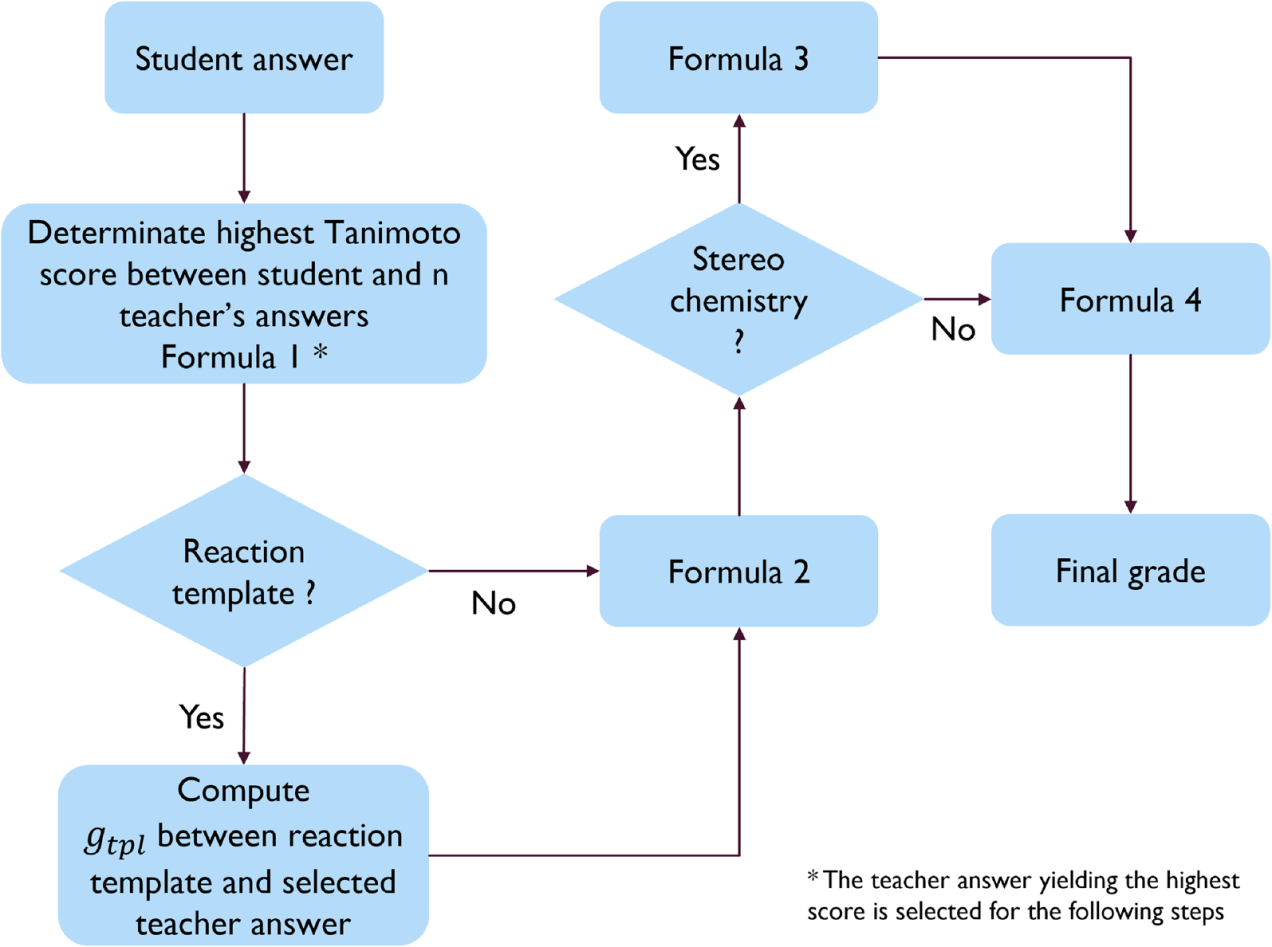
Decision network summarizing how the grade is computed according to different user cases scenario

As a function of the type of teacher’s question, several scenarios of grading are considered (Fig. 6).

(a) Reaction template is not given (e.g., “Prepare equation for Diels–Alder reaction between butadiene and ethylene”). In this case, the grade *g*_*sim*_ is the maximal Tanimoto similarity between the students’ answers and the 1 to N teachers’ answers (Formula 1).

1 $${T}_{s}={g}_{sim}=\frac{A\bigcap B}{A\bigcup B}=\frac{{\sum }_{i}{A}_{i}{B}_{i}}{{{\sum }_{i}{A}_{i}}^{2}+{{\sum }_{i}{B}_{i}}^{2}-{\sum }_{i}{A}_{i}{B}_{i}}$$

(b) If several alternative answers are possible (see Fig. 5), a maximal similarity score is taken as *g*_*sim*_. If a teacher employs a “reaction template”, the algorithm will assess the similarity between the template and the full reaction equation of teacher’s answers that yielded the maximal Tanimoto similarity (*g*_*tpl*_) even if a student gives no answer at all. In order to take the “reaction template” into account, a final score is modified according to formula (2) where *g*_*sim*_ is a similarity score related to the student’s answer.

2 $$g_{rest} = \left\{ {\begin{array}{*{20}c} {\frac{{\left( {g_{sim} - g_{tpl} } \right)}}{{\left( {1 - g_{tpl} } \right)}},} & {if ^{\prime\prime}reaction \,template^{\prime\prime}} \\ {g_{sim} ,} & { if \,no ^{\prime\prime}reaction \,template^{\prime\prime}} \\ \end{array} } \right.$$

(c) If stereochemistry is not requested by the teachers, the computed grade *g*_*rest*_ is sent back to Moodle. Otherwise, the InChI [15] strings are used to compare stereo centers (R/S or Z/E) of each reactant and product from the students and teachers reactions. The grade *g*_*rest*_ will be computed as the proportion of correctly drawn stereo centers (“#CorrectStereoCenter”) over the total number of stereo centers in the reaction (“#TotalStereoCenter”), and sent back to Moodle (Formula 3), in the same way as for the *Molsimilarity* module.

3 $$g_{rest } = \left\{ {\begin{array}{*{20}l} {\frac{\# \, Correct \, Stereo \, Center}{{\# \,Total \, Stereo \, Center}},} & {if \, similarity \, score, g_{sim} = 1} \\ {0,} & {if \, similarity \, score, g_{sim} \ne 1} \\ \end{array} } \right.$$

It should be noted that application of InChI strings to compare stereo implies that compared chemical structures may only differ on the stereochemistry. Indeed, a change in any part of a chemical function may alter its priority level to assess the stereochemistry label. Because of this, if the similarity score *g*_*sim*_ is not equal to 1 and the stereochemistry is required in the grading process, a *g*_*rest*_ of 0 is returned to Moodle.

Once the stereochemistry assessment is performed, the score *g*_*rest*_ is sent back to Moodle, where the final grade *g* is calculated according to Formula 4. This formula introduces the user-defined parameters *t* and α. These parameters allow to modulate the softness of the grading: for small values of α, more errors will be tolerated while large values will deteriorate the grade for any deviation from the expected answer. If the computed grade is below the cutoff parameter *t*, no points are given to the question. By default,* t* is equal to 0 and has a range going from 0 to 1, and *α* is equal to 1 and has a range going from 0.1 to 10. Both parameters are set by the teachers while preparing the question and each question can have different values of these parameters. Finally, the general feedback is displayed to the students, containing the expected answer, as well as the specific feedback if *g* < 1.

4 $$\text{g}= \left\{\begin{array}{l}{({\text{g}}_{rest})}^{\alpha }, if ({{\text{g}}_{rest})}^{\alpha } \ge \text{t} \\ 0, \,\,otherwise\end{array}\right.$$

Equivalent AAM (for instance, by permutation of the atom numbering) are accepted as correct. For this reason, the expected answer displaying the solution proposed by the teacher and the student answer may appear visually different while being equivalent and maximally graded. This display can be confusing to some students.

## Conclusion

The development of automated tools for online chemistry homework assignments has become increasingly important, especially in the context of distance learning and the need for continuous assessment.

This article proposes a new approach that expands the scope of automated correction from individual molecules to entire chemical reactions using the concept of Condensed Graph of Reaction (CGR). By utilizing CGRs, atom mapping, and substructural fragments, the proposed tool enables the correction and assessment of students answers at a holistic level, capturing the complexity of chemical reactions.

The incorporation of AAM in the students’ answers not only eases the grading process but also serves as an educational exercise, allowing students to gain a deeper understanding of chemical reactions at the atomic level.

The implementation of the tool involves a user-friendly interface and utilizes Chemdoodle Web Component to draw reactions. The grading process involves creating the CGRs, encoding them using ISIDA fragment descriptors, and computing pairwise Tanimoto similarity between students’ and teachers’ answers.

This work could be improved by adding several features. It should be possible in a next update, to make the use of AAM optional. The question could then be to draw a chemical reaction whatever the AAM is. The stereochemistry correction information could be used to build specific feedback for the students. This feedback would categorize answers as identical to the correct answer, a constitutional isomer, a diastereomer, an enantiomer, or introducing ambiguity, offering much richer insights to students. The University of Strasbourg is organizing hackathon and is sharing experience through meetings such as the Moodle Moot. Indeed, the use of a new tool needs some training. Specific documentation designed for teachers will also be shared with the community.

The proposed tool offers several advantages, including the ability to handle multiple correct answers, the option to provide initial reaction templates, and the generation of both general and specific feedback for students. By combining the strengths of open-ended and closed-ended question types, the tool provides objective, efficient, and knowledge-based assessment.

## Data Availability

No datasets were generated or analysed during the current study.

## Materials availability

All material is provided free of charge under GNU GPL v3 licence. All material is available either on the Moodle store (https://moodle.org/plugins/qtype_reacsimilarity) or the Git of the project (https://github.com/Laboratoire-de-Chemoinformatique/moodle-qtype_reacsimilarity).
